# CLIMATRIAL: applicability and adaptability of a novel method to quantify the carbon footprint of a South African clinical trial

**DOI:** 10.1186/s13063-025-08903-w

**Published:** 2025-06-18

**Authors:** Nandi Louise Siegfried, Lesley-Ann Erasmus-Claassen, Jessica Griffiths, Lisa Fox, Paula R. Williamson

**Affiliations:** 1 Mental Health, Alcohol, Substance Use and Tobacco Research Unit, South African Medical Research Council, Francie Van Zijl Drive, Parow Valley, Cape Town, 7501 South Africa; 2 Clinical Trials and Statistics Unit, The Institute of Cancer Research, London, UK; 3 Department of Health Data Science, MRC-NIHR Trials Methodology Research Partnership, University of Liverpool, Liverpool, UK

**Keywords:** Carbon emissions, Trials, Carbon tracking tools

## Abstract

**Background:**

No formal assessment of a method to measure the carbon emissions from a clinical trial has been undertaken in the low- and middle-income country setting. We aimed to evaluate the UK-based National Institute of Health Research (NIHR) method for tracking trial emissions for applicability and adaptability to the South African context. Objectives included to (1) identify gaps in trial activity data, (2) locate local emission factor sources, (3) quantify emissions and (4) investigate modifications required to ensure the method was locally applicable.

**Method:**

We adopted an application and implementation approach. We established a formal stakeholder structure focused on sustainable clinical trials to guide and support our approach. We selected a large cluster-randomised trial of a health service delivery intervention conducted across multiple urban and rural sites as an exemplar typical of local conditions to test the NIHR method. We created a trial process map outlining ten recommended activity stages for carbon emissions and an Excel workbook to calculate emissions for each stage. We prioritised calculations of those activities for which we had the most complete data: paper usage and printing, local travel between sites, and electricity consumption at the trial head office and at trial sites. We extracted activity data from organisational financial instruments.

**Results:**

The study took place between December 2023 and March 2024. We identified a lack of publicly available local emission factor sources. Paper usage and printing activities took place at trial set-up and during intervention delivery and emitted 2274.88 kgCO_2_e. Field staff travel between trial sites during intervention delivery and follow-up resulted in approximately 80,000 km travelled between 2016 and 2019 contributing 17,891 kgCO_2_e. Electricity consumption was based on full-time equivalent staff and yielded 12,515 kgCO_2_e during the 4-year period. We observed large differences between UK and available SA emission factors with SA emission factors far higher than those in the UK.

**Conclusion:**

We found that with minor modifications, the NIHR guidance is applicable to the SA context. It is a highly adaptable framework permitting tracking of activities across trial stages. A lack of locally available emission factors reduces accuracy and emission results should be viewed as indicative.

## Background

The harmful effects of climate change on human health are now widely acknowledged with greenhouse gas emissions responsible for increasing pollution, resultant global warming and extreme weather phenomena [1]. Healthcare services are estimated to account for 4.4% of global greenhouse gas emissions (based on 2014 data) [2]. Most healthcare interventions have yet to be fully analysed with a climate lens including the need to reduce emissions arising from medical research [3]. Clinical trials, which provide essential evidence on the benefits and harms of health interventions, are conducted primarily within healthcare services. This creates a duality for investigators: trials generate a public good by informing an evidence base of effective health-related interventions while simultaneously potentially adding to public harms through greenhouse gas emissions. The development and implementation of methods to reduce and/or mitigate greenhouse gas emissions during the conduct of a trial is gaining visibility, but data from carbon emissions remains limited to publications largely from the UK [4] and industry [5, 6]. To date, no formal assessment of carbon emissions has been undertaken in a trial conducted in the South African publicly funded healthcare sector.

Beyond justice and rights-based imperatives to reduce trial emissions, funding agencies and organisations may request trial investigators to monitor, quantify and/or reduce trial emissions. Without validated tools to identify emission activities and measure data reliably, South African trial investigators have no means to do this. In this study, we aimed to apply the UK National Institute of Health Research-funded method and guidance to calculate the carbon footprint of a clinical trial (known as the NIHR method hereafter) [4] to a South African trial and evaluate it for local applicability and adaptability beyond the UK.

## Methods

Our evaluation adopted an application and implementation approach. Our objectives were [1] to retrospectively quantify the carbon footprint of a South African clinical trial using the NIHR-funded method and guidance and [2] to identify gaps in trial data and local emission factor sources to inform local applicability and adaptability of the method.

At study outset, we established a formal stakeholder structure within our organisation, the CLIMATRIAL Sustainable Trials Working Group, to support our approach and to guide the implementation of the study. Prior to the study, the local team attended training in the application of the method via the NIHR and Trials Methodology Research Partnership-funded Carbon Footprinting Drop-in Clinics [7] as well as receiving support when required on an ad hoc basis. We selected Project MIND as a typical local trial to serve as exemplar [8]. MIND was a three-armed, cluster-randomised controlled trial of mental health service delivery interventions conducted in 1340 patients from 24 primary care clinics in both urban and rural settings over a 4-year period between 2016 and 2019, thereby providing complexity and multiple locations to yield comprehensive data activities for carbon tracking. Advantageously, we had previously collated secondary and source data and created a trial life cycle diagram for Project MIND when conducting an evaluation of its cultural competency [9].

**Fig. 1:**
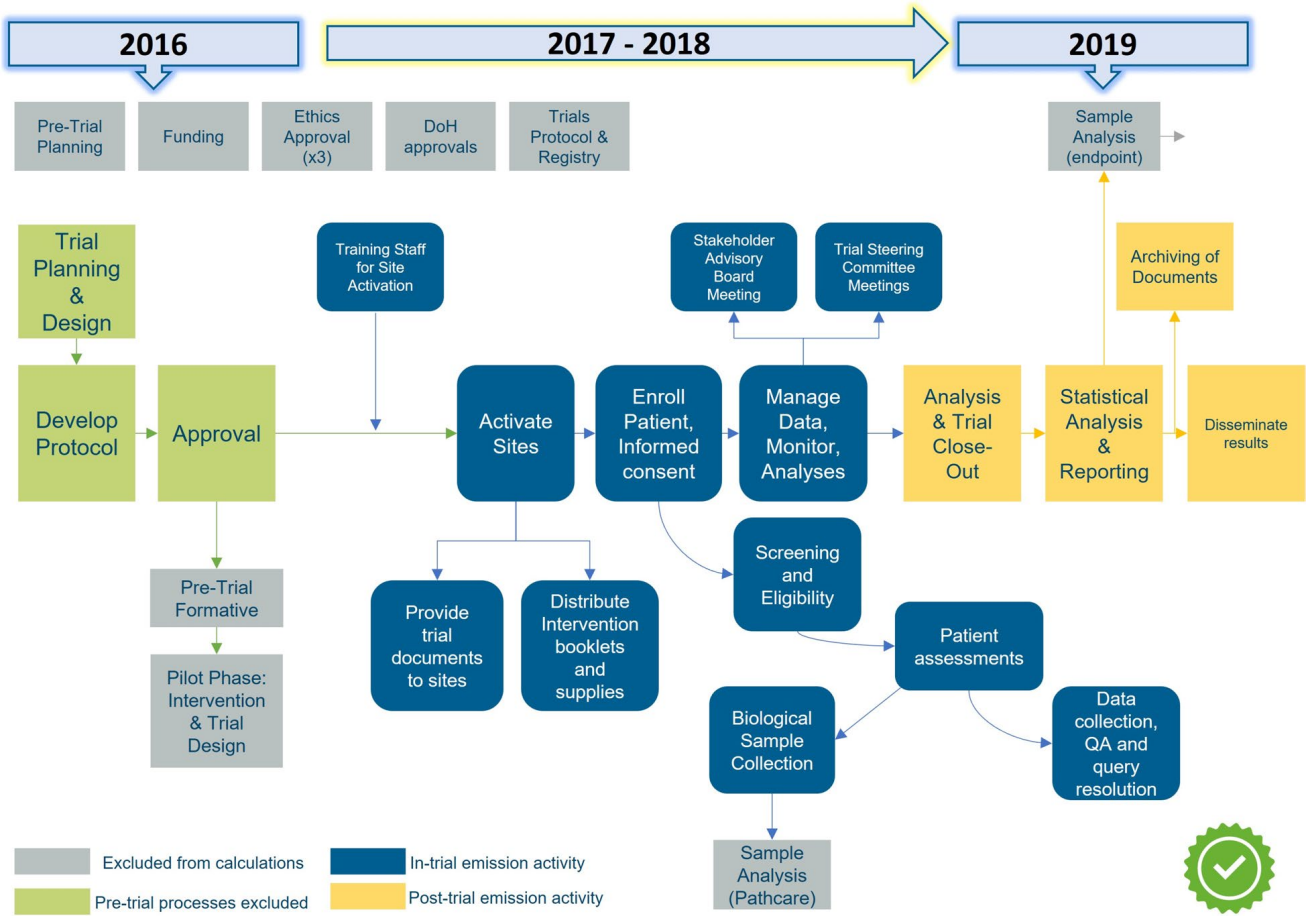
CLIMATRIAL Project MIND

We revised the trial life cycle diagram to create a trial process map outlining the ten recommended activity stages for carbon emissions: trial set-up; trial unit emissions (includes energy and heating used in research premises, trial staff commuting and statistical analysis); trial-specific meetings and travel; treatment intervention; data collection and exchange; trial supplies and equipment; trial-specific patient assessments; samples; laboratory; and trial close out. Where trial activities comprise standard of care healthcare processes, these are not included within the trial-specific footprinted activities. Other activities excluded from the trial footprint include manufacture of intervention (e.g. investigational medicinal products (IMPs)) and regulatory approval, as these activities are not within the control of trialists nor will data be accessible to trialists. The NIHR-funded method and guidance accounts only for the carbon footprint of a trial, and no other environmental impacts such as land and water use, or potential economic and social trade-offs [4] (see Fig. 1).

We created an Excel workbook comprising worksheets for each activity stage to calculate carbon emissions (the ‘carbon footprint’) according to the formulae as per the NIHR-funded method [4]. Carbon footprint is a measure of the carbon emissions from a process, product or organisation and is calculated in carbon dioxide equivalent (CO_2_e). The NIHR-funded method limits measurement to carbon footprinting because this is the impact that trialists designing and conducting clinical trials can influence directly [4]. Methods for calculating carbon footprint can be broadly categorised as bottom-up (lifecycle assessments of specific processes usually requiring extensive data) or a top-down approach (appropriate for sectoral level input-output evaluation) [10]. The bottom-up approach adopted in the NIHR-funded method calculates the carbon footprint of the trial by multiplying trial activity data by emission factors [4]. We planned to source local emission factors where available to replace UK emission factors in the calculations and contacted individuals and institutions and searched the internet for publicly available local emission factors. We used unaltered UK factors in our calculations in lieu of local factors when we were unable to source these.

We requested access to the SAMRC organisational financial ledger which documented all expenses for Project MIND over the full trial lifecycle. We re-categorised the activities that resulted in expenses to map to the related trial emissions activity; where possible, we noted at which stage of the trial the activity took place. If overall costing data was uncertain, we accessed discrete invoicing instruments for granular details. For example, costs for paper were not static over time likely reflecting changing procurement practices as well as inflation, and we thus required invoice details to quantify the actual amounts of pages procured. Local car travel for trial team site visits was not available as mileage data directly and was recorded in the ledger as the monetary amount reimbursed for travel using a revenue-approved allowance. This required adjustment for annual changes in the national mileage tax reimbursement rates before we could calculate kilometres travelled. For obtaining information on some activities, we relied on the personal knowledge of the trial project manager, e.g. for estimating the square meterage of dedicated trial space in the clinic environment and for determining full-time equivalent values permanent staff contributed to the trial.

## Results

### Emission factors

No single government repository for emission factors exists in South Africa. Through web-searching, we identified a publicly available 2021 grid emission factor for the electricity system published by the Department of Forestry, Fisheries and the Environment [11] and a carbon emission factor for paper production used by a local university in its carbon footprinting assessment [12]. Table 1 compares South African factors with those of the UK.

**Table 1:** Comparison of UK and available South Africa emission factors

	UK	South Africa
Paper emission factor (kgCO2e/kg paper)	0.919 (2021) [4]	2.044 (2019) [12]
Cardboard emission factor (kgCO2e/kg paper)	0.821 (2021) [4]	2.044 (2019) [12]
Electricity grid emission factor (kgCO2e/kWh)	0.273 (2021) [4]	1.013 kgCO2e/kWh (2021) [11]

### Trial activity emissions

After noting the lack of local emission factors and the complexity of extracting necessary activity data from financial ledgers, we were advised by the CLIMATRIAL Sustainable Trials Working Group, to prioritise three focal activities for which we had the most complete data, viz. [1] paper use, [2] local car travel to and from clinic sites by the trial management or field team and [3] electricity consumption at the trial head office and at clinic sites. These activities were considered to best reflect typical data to inform the local applicability of the NIHR-funded method. Project MIND evaluated a service delivery intervention requiring training of community health workers using paper-based education materials before delivering a screening and counselling intervention. Each trial site made use of cardboard folders with printed prompter pages to compile data related to the delivery of the intervention. The trial did not evaluate an IMP and therefore activities did not include medicine packaging, couriering or significant cold chain requirements. Biological samples obtained from participants were transported and stored as part of routine laboratory services except for a single rural clinic which stored samples overnight prior to collection by a courier. We did not factor in the electricity emissions for the cold storage from the single refrigerator.

Staff travelled regularly between the clinics which were spread across the Western Cape Province, an area of approximately 129,462 km^2^ [13] with the furthest trial clinic 325 km from Cape Town. The province is not well-serviced by public transport routes. This reflects the typical geographic challenges posed by multi-site trials in South Africa.

Factors rounded to 3 decimal places. UK factors were used in the Griffiths et al. [4] trial case study and are from https://www.gov.uk/government/publications/greenhouse-gas-reporting-conversion-factors-2021.

South African paper and cardboard factors were obtained from a local institutional carbon footprinting report [12] derived from a local paper manufacturer; energy was from a government report [11].

#### Paper usage and printing

Paper usage and printing activities took place during the trial set-up and intervention delivery stages and emitted 2274.88 kgCO_2_e. During trial set-up, printed training materials on screening and counselling for depression and alcohol and substance use were prepared for distribution to clinic nursing staff, two cardboard hanging folders were created for each trial participant, and participant reimbursement vouchers (monetary amounts printed on pages in booklets) were procured. At the intervention delivery stage, disease-specific self-care booklets were printed and disseminated to each trial participant.

#### Travel between clinic sites

Trial staff frequently travelled between clinics for quality assurance check-ins and interventionist debriefing visits, resulting in a total of 80,000 km over the 4-year period contributing to 17,891 kg CO_2_e. One international flight to the UK contributed 3948 kg CO_2_e and a local flight between Cape Town and Johannesburg contributed 1173 kg CO_2_e.

#### Electricity consumption at the clinical trial head office and at clinic sites

Full-time equivalent (FTE) research staff based at the SAMRC institutional building (the clinical trial head office) and at the clinic sites ranged between 3.7 and 4.75 FTE annually contributing 12,515 kgCO_2_e in electricity use in total over the 4-year period. During this time period, South Africa experienced several months of power outages (‘load-shedding’), usually for 2-h periods but sometimes up to 6 h daily. During these times, building and clinic electricity was provided by diesel generators. This was not factored into the calculations.

## Discussion

Retrospective application of the NIHR method of carbon footprinting to a South African trial presented specific challenges due to the lack of publicly accessible local emission factors and complexity in extracting specific trial activity data from institutional operations-related records. Nonetheless, the NIHR-funded method proved adaptable providing a flexible framework for calculating overall kgCO_2_e for activity categories across trial stages instead of tracking trial activity within each of the ten pre-specified trial stages. Carbon emissions calculated in this way provide indicative measurements of within-trial emissions and permit identification of targeted activities where future trial investigators can reduce and mitigate emission-producing activities.

According to the Greenhouse Gas Protocol, carbon accounting is broken down into scope 1 (direct emissions produced by an organisation), scope 2 (indirect energy emissions usually purchased electricity) and scope 3 (other indirect emissions) [14]. In the trial context, scope 3 emissions are a consequence of the activities of the trial, but occur from sources not owned or controlled by the organisation. In Project MIND, production of purchased material such as paper and employee travel to sites are examples of scope 3 emissions. In MacKillop et al.’s evaluation of three industry trials, they observed a large disparity for scope 3 emissions between determining these from financial proxies compared with collecting and using activity-based data based on supply chain [5]. Our calculations relied on reported supply chain data, possibly improving the output accuracy, but at best, all measurements should be viewed as indicative and useful primarily for comparison and planning purposes. Scope 2 emissions relate to the use of electricity and energy at the SAMRC and clinic settings and are likely an under-estimation given the aforementioned practice of transferring to diesel generation during country-wide load-shedding. This is likely to be relevant to other low- and middle-income countries where power supply is unpredictable. Omission of carbon calculations related to diesel generation will affect the local adaptability and accuracy of the NIHR method in such settings and exemplifies the need to identify applicable country-specific activities and emission factors.

Available local emission factors were largely lacking and we were disappointed to observe that no government repository of local emission factors exists, unlike in the UK. This likely reflects the relatively under-developed application of carbon accounting as well as the fragmented energy sector in South Africa [15]. Comparison of South African to UK emission factors clearly demonstrates the effect of the South African coal-based energy sector with factors far greater than those in the UK. Similar to many countries, both rich and poor, South African government commitment to fossil fuels remains high [16]. Recent government commitments to a just transition to a low carbon economy aims to ensure renewable energy security [17]. Paid subscriptions for modelled South African factor data is available but the NIHR-funded method intends to be open-access and we therefore selected to include only those local factors freely available to us and to use UK factors as proxies where necessary. While this remains a current limitation rendering our results indicative rather than accurate, the development of the NIHR method tailored to local needs will permit more accurate local emissions factors to be substituted and included into calculations as these become available.

In our application of the NIHR-funded method to Project MIND, which evaluated a non-IMP, we have noted the necessity to further expand the current guidance to include more non-IMP trials as worked examples. In addition, language and examples should accommodate and be inclusive of all types and settings of trials including schools and community centres, to encompass prevention and health promotion trials conducted in non-clinical settings as well as treatment trials conducted in the homes of participants. We believe this will enhance its adaptability and generalisability beyond the clinical environment.

The first step to reducing the carbon footprint of a planned clinical trial is to reliably measure its potential footprint and identify carbon hotspot activities [4]. In our retrospective application of the NIHR-funded method to Project MIND, we were unable to identify hotspots within all ten trial stages due to lack of data. However, our findings did reveal the extent of emissions from paper, printing and staff travel between trial sites. On the face of it, it would seem relatively simple to reduce or eliminate these emissions entirely. Progress from paper-based to digital training materials and digital participant records is considered inevitable, but a recent editorial in the *Lancet Digital Health* posed the question of whether wider adoption of digital health technologies will mitigate or exacerbate the issue [18]. It is not a given that the emissions required to develop and run technological advances are necessarily less than those generated by paper production. The editorial goes on to recommend that standardised methods and tools are needed to assess the environmental effects of digital health technologies. Yet, trials planned in the South African setting may need to rely on paper systems for the foreseeable future as the transition to digital records and patient booklets continues to be challenging. This is especially true in rural and peri-urban settings, where many clinics have limited or no computer access, and Wi-Fi is slow or non-existent.

Travel between clinic sites was a significant driver of carbon emissions in Project MIND. The location of multiple sites based both in urban and rural areas is typical of many trials in South Africa. Sparsely inhabited geographic regions are poorly served by public transport routes necessitating use of private vehicles to cover large distances, often single-occupant vehicle use. We envision that this activity can be considerably reduced in future trials. The COVID-19 pandemic normalised virtual meetings and as such, trial investigators requiring regular contact points with clinic staff can replace in-person visits with virtual options. Where scarcity of computers in clinics precludes this, trial budgets should include provision for tablet or mobile phones for clinic staff with linked internet connectivity coverage for the duration of the trial. Continuing to hold in-person training meetings where a trainer’s single travel emissions may be justifiable if less than those produced by training delivered to groups of clinic staff via multiple digital means. Recruitment prediction and monitoring tools should be an essential component to trial planning to better coordinate and plan travel networks to sites when clinics may require an in-person inspection or assistance [19].

Lastly, as healthcare professionals, we recognise that our skills and knowledge in the field of carbon accounting are emergent. In the past, we have demonstrated the value of collaboration between clinicians and engineers to optimise our respective skillsets for the development of practical and impactful solutions to methodological and trial management challenges [19]. It is clear that opportunity exists for further intersectoral collaborations in research and solution development within the greener trials environment. We wholeheartedly support the *Lancet Digital Health* editorial recommendation to foster interdisciplinary collaborations with multiple stakeholders including patients, clinicians, environmental scientists and computer scientists, with the ultimate aim to reduce the carbon impact of healthcare and related research and to improve the health of the planet and its people [18].

## Data Availability

No datasets were created during this study. The spreadsheet with trial activity and calculations is available on request. The organisational financial instruments related to costs of the trial are not able to be shared.
